# Deep Sequencing Analysis of HCV NS3 Resistance-Associated Variants and Mutation Linkage in Liver Transplant Recipients

**DOI:** 10.1371/journal.pone.0069698

**Published:** 2013-07-29

**Authors:** Mariana E. Kirst, Eric C. Li, Cindy X. Wang, Hui-Jia Dong, Chen Liu, Michael W. Fried, David R. Nelson, Gary P. Wang

**Affiliations:** 1 College of Medicine, Department of Medicine, Division of Infectious Diseases and Global Medicine, University of Florida, Gainesville, Florida, United States of America; 2 College of Medicine, Department of Pathology, College of Medicine, University of Florida, Gainesville, Florida, United States of America; 3 Department of Medicine, Division of Gastroenterology, University of North Carolina Chapel Hill, North Carolina, United States of America; 4 College of Medicine, Department of Medicine, Division of Gastroenterology, University of Florida, Gainesville, Florida, United States of America; Saint Louis University, United States of America

## Abstract

Viral variants with decreased susceptibility to HCV protease inhibitors (PIs) occur naturally and preexist at low levels within HCV populations. In patients failing PI monotherapy, single and double mutants conferring intermediate to high-level resistance to PIs have been selected *in vivo*. The abundance, temporal dynamics and linkage of naturally occurring resistance-associated variants (RAVs), however, have not been characterized in detail. Here, using high-density pyrosequencing, we analyzed HCV NS3 gene segments from 20 subjects with chronic HCV infection, including 12 subjects before and after liver transplantation. Bioinformatics analysis revealed that Q80 substitution was a dominant variant in 40% of the subjects, whereas other RAVs circulate at low levels within quasispecies populations. Low frequency mutation linkage was detectable by Illumina paired-end sequencing in as low as 0.5% of the mock populations constructed from *in vitro* RNA transcripts but were uncommon *in vivo*. We show that naturally occurring RAVs are common and can persist long term following liver transplant at low levels not readily detectable by conventional sequencing. Our results indicate that mutation linkage at low levels could be identified using the *Illumina* paired-end approach. The methods described here should facilitate the analysis of low frequency HCV drug resistance, mutation linkage and evolution, which may inform future therapeutic strategies in patients undergoing direct acting antiviral therapies.

## Introduction

Hepatitis C virus (HCV) infects over 180 million individuals worldwide and is the leading cause of liver transplantation due to cirrhosis and hepatocellular carcinoma [1]. Historically, interferon-based regimen with ribavirin is the gold standard for the treatment of chronic HCV infection, but the rate of sustained virologic response (SVR) has been suboptimal. Remarkably, the addition of NS3/4A protease inhibitor to peginterferon-ribavirin improves the SVR rate substantially in both treatment naïve and treatment experienced individuals [2]–[9]. With a pipeline of direct acting agents (DAAs) in development, there is tremendous enthusiasm for HCV therapeutics. While data for new protease inhibitors are encouraging, resistance to this and other classes of drugs may become an important consideration, especially in anticipation of interferon-free regimens in the coming years.

Resistance-associated variants (RAVs) to NS3/4A protease inhibitors have been identified in both *in vitro* studies and clinical trials [10], [11]. In treatment-naïve patients, naturally occurring dominant RAVs are common [12]. The quasispecies nature of HCV raises the concern that viral swarms may harbor preexisting mutations at low frequency not readily detectable by conventional genotyping methods, which may influence treatment outcome. Indeed, mathematical modeling of HCV replication suggests that RAVs preexist, which can emerge rapidly under selective drug pressure [13], [14]. The presence of preexisting RAVs is further supported by the observation that RAVs are selected rapidly in subjects receiving protease inhibitor monotherapy [10], [11], typically within days of initiating DAAs. Importantly, lessons from the HIV field indicate that preexisting drug resistant variants at low frequencies could contribute to treatment failure [15], and thus genotypic resistance testing is now the standard of care prior to initiating antiretroviral therapy. For HCV, the prevalence of dominant, naturally occurring RAVs has been previously reported [12], [16]–[18]. However, the abundance, mutation linkage and evolution of RAVs that circulate at low frequencies have not been characterized in depth in patients undergoing liver transplantation. A detailed examination of resistance profiles is important for identifying clinically relevant drug resistance variants and optimizing strategy to improve treatment outcome.

Studies of low frequency HCV variants have long been hampered by the lack of sensitive sequencing methods. New sequencing technologies, such as Roche/454 pyrosequencing and Illumina sequencing, have made it possible to deeply sequence a larger number of samples simultaneously. The use of these technologies has led to sensitive detection of low abundance mutations in HIV and HBV quasispecies [19], [20], and the analysis of HCV viral dynamics and transmission bottlenecks [21]. Here, we present the results of sequencing barcoded PCR amplicons to quantify variants associated with NS3 resistance from 20 subjects with chronic HCV infection, including longitudinal samples from 12 liver transplant recipients. We show that naturally occurring, low frequency RAVs are common in chronic HCV, and can persist long term following liver transplantation. We also addressed the question of whether linkage between mutations far apart on the same HCV genome could be quantified using a modified Illumina paired-end sequencing approach. We used mock *in vitro* transcribed RNA communities to show that the paired-end approach could identify linked variants at two ends of long amplicons. Although linkage of pre-existing mutational variants was uncommon in our treatment-naïve cohort, the paired-end approach should be useful during direct antiviral therapy and generally applicable to linkage analysis in other genomic loci of HCV or other viruses. The methods described here should facilitate longitudinal analyses of RAVs *in vivo* and provide a framework for future studies on the impact of preexisting RAVs on treatment outcome using DAAs.

## Methods

### Ethics statement

Serum samples before and after liver transplantation were collected at the University of North Carolina Liver Center under a University of North Carolina at Chapel Hill Institutional Review Board-approved protocol with written informed consent from all participants. Archived clinical samples for chronic HCV were obtained at University of Florida under a University of Florida Institutional Review Board protocol approved for a waiver for Informed Consent in accordance with 45 CFR 46.116(d) under research category #5 for research involving materials (data, documents, records, or specimens) that have been collected, or will be collected solely for nonresearch purposes (such as medical treatment or diagnosis). All samples were from subjects who were protease inhibitor treatment naïve.

### Amplification of NS3 gene segments

**Figure 1:**
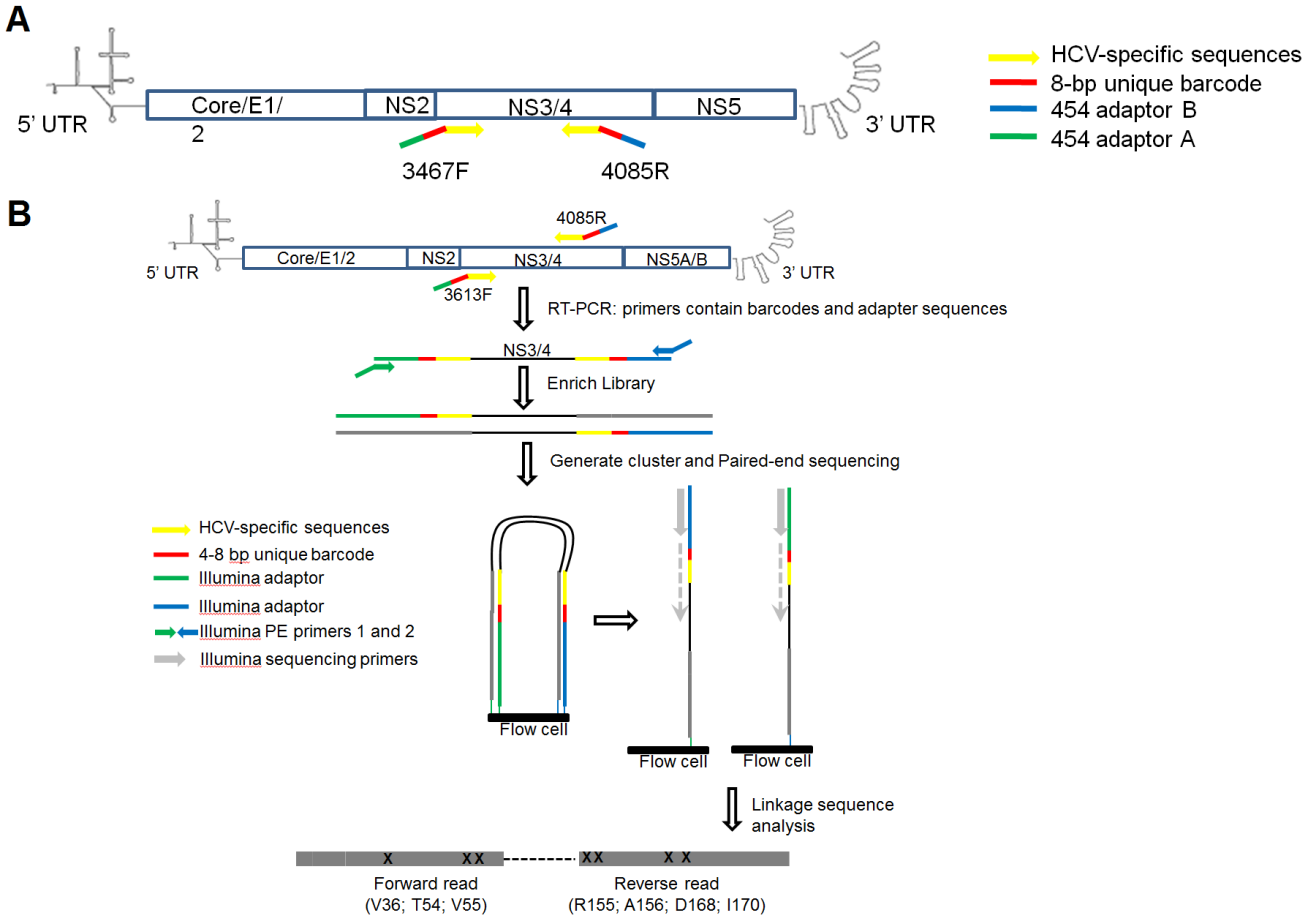
Deep sequencing strategy to determine abundance and mutation linkage of NS3 RAVs. (A) Roche/454 pyrosequencing. Primer binding sites for 454/Roche pyrosequencing primers are shown in yellow. The pyrosequencing primers are composites containing the required sequences for the Roche/454 titanium chemistry procedure at 5′ end (blue and green), a unique 8-base DNA barcode that indexes each sample (red), and HCV-specific primer sequences at the 3′ end (yellow). (B) Illumina paired-end sequencing. Partial NS3 gene segments were first amplified using gene-specific primers that contain HCV-specific sequences (yellow), a barcode sequence unique to each sample (red) and partial adapter sequences (green and blue). Amplified fragments were tailed with flow cell adaptors (green and blue arrows), then subjected to paired-end sequencing using the standard Illumina paired-end protocol. This protocol will generate non-overlapping forward (100 nt) and the reverse (100 nt) reads from each cluster, providing precise long-range positional and sequence information. After trimming and quality control, the paired-end reads allowed linkage analysis between V36-V55 and R155-I170. Sequences of all primers used in (A) and (B) are listed in Supporting Information S1.

Viral RNA was extracted from plasma, and quantified by quantitative RT-PCR using primers specific to highly conserved 5′ UTR of the HCV genome (Supporting Information S1). For pyrosequencing, NS3 gene segments of approximately 600 nt (corresponding to coordinates 3342 to 3951 on the H77 genome, accession NC_004102) were amplified using HCV-specific primers that contained the required adaptor sequences for the Roche/454 titanium chemistry procedure and a unique 8-bp barcode that indexed each sample, which allowed multiplex pyrosequencing. For Illumina sequencing, the gene-specific primers contained unique 4 to 8 bp index sequence and Illumina PE adapters (Figure 1 and Supporting Information S1). Control RNA of known sequence was generated by *in vitro* transcription of linearized plasmid containing a T7 promoter and full-length H77C genotype 1a sequence. The *in vitro* control transcripts were subjected to identical experimental procedures including RT-PCR and pyrosequencing as patient-derived viral RNA (Supporting Information S1).

### Roche/454 pyrosequencing

RT-PCR amplicons were gel purified, pooled, and subjected to bidirectional pyrosequencing using the Titanium chemistry on the Roche/454 GS-FLX platform. Pyrosequence reads were filtered using the following quality control criteria: (i) an exact match to barcode and primer sequences, (ii) >360 bases in length for forward reads; >290 bases for reverse reads, and (iii) no ambiguous bases (Ns) (Supporting Information S1). Forward and reverse reads were trimmed to ∼337 and ∼264 bases, respectively, then aligned to H77C reference sequence using global multiple sequence alignment. The codons associated with resistance to protease inhibitors were identified. Of the reads that contained codon-changing nucleotide substitutions, pairwise sequence alignments were performed followed by manual inspection of the aligned sequences. Technical error rates were determined using *in vitro* control transcripts of known NS3 sequences that were amplified and sequenced in parallel. To distinguish authentic variants at drug resistance sites from technical artifacts, position-specific background error rate was calculated to define authentic drug resistance mutations using a chi-square test at p≤0.05. The forward pyrosequence reads were used to form operational taxonomic units (OTUs) for subsequent quasispecies diversity analysis.

### Quasispecies diversity analysis

Forward 454 pyrosequence reads (corresponding to coordinates 3342 to 3674 on the H77 genome, accession NC_004102) were clustered into operational taxonomic units (OTUs) with a 97% identity threshold (accepting nine differences over 300 nt), excluding chimeras and sequences represented by singletons and doubletons. As described previously [21], we chose a 3% difference to cluster reads into OTUs as a reasonable compromise for trying to maintain some of the rare authentic variability while minimizing error-induced variability. For each OTU cluster, reads were aligned to generate a consensus sequence (∼337 bp), which was used to represent the dominant sequence for each quasispecies (or OTU) and for phylogenetic analysis. Phylogenetic trees were constructed using the UPGMA method with either Geneious or the Phangorn package in R [22]. Shannon index values were calculated to determine quasispecies diversity (Supporting Information S1). All sequence datasets have been deposited in the NCBI Sequence Read Archive (SRA) under accession no. SRA059141.

### Mutation linkage analysis by Illumina paired-end sequencing

Because the forward and the reverse 454 reads (following trimming of barcode and primer sequences) did not overlap to allow reconstruction of haplotypes for linkage analysis, we applied the Illumina paired-end technology to determine mutation linkage. Partial NS3 gene segments (∼460 nt) were amplified using gene-specific primers that contain a barcode sequence unique to each sample and partial sequences for the adaptor (Supporting Information S1). Therefore, linkage between paired-end reads was determined based on the assigned barcodes and the sequences coordinates in the illumine flowcell. Amplified fragments were gel-purified, pooled at equimolar concentrations, and tailed with flow cell adaptors (Figure 1). The enriched library was quantified using Kapa Library Quantification kit (Kapa Biosystems, Woburn, MA) and subjected to standard Illumina paired-end sequencing at 2×100 bp on Illumina Genome Analyzer IIx at the University of Florida ICBR sequencing core. Paired-end reads were processed using the following criteria: (i) an exact match to barcode and primer sequences; (ii) no ambiguous bases; and (iii) both forward and reverse sequences pass all quality steps (no reads with unknown ‘B’ quality scores and no reads that failed Illumina quality check ‘0’). The filtered, trimmed reads were aligned to H77C reference sequence, and the codons associated with PI resistance were identified and the mutation frequency calculated.

## Results

### Patients and samples

We performed direct Sanger population sequencing and Roche/454 pyrosequencing of partial NS3 gene fragments on 55 serum samples from 20 PI treatment naive subjects with chronic HCV infection. These included samples from 8 subjects with chronic HCV infection and 47 longitudinal HCV samples from 12 liver transplant (LT) recipients, in whom up to 5 samples pre- and post-LT were analyzed (Table 1). We also performed Illumina paired-end sequencing to assess mutation linkage in selected samples. All 20 subjects harbored genotype 1 virus (14 GT1a and 6 GT1b), as determined by NS5B gene sequencing [23]. All 12 liver transplant recipients received immunosuppressive regimen post-LT.

**Table 1:** Demographics, viral load, and time points of the samples used in this study.

Subject ID	Age	Gender	Ethnicity	Genotype	Time point (days)	Viral Load (IU/mL)	Immunosuppression Regimen
A	41	Male	Caucasian	1a	N/A	1,192,920	N/A
B	56	Male	Hispanic	1a	N/A	1,636,980	N/A
C	51	Male	Caucasian	1a	−973	64,000	
					−133	N/D	
					68	N/D	Prograf
					371	>700,000	Prograf
D	44	Male	Native American	1a	−113	N/D	
					380	357,000	Tacrolimus
					810	2,510,000	Prograf
E	46	Male	Caucasian	1a	−66	N/D	
					57	>700,000	Prograf
					746	418,000	Prograf
F	54	Male	African American	1a	−284	293,000	
					99	N/D	Prograf/Prednisone
					1163	>5,000,000	Prograf
G	50	Male	Caucasian	1a	−840	N/D	
					−70	125,000	
					534	439,000	Prograf
					1162	1,070,000	Prograf
H	48	Male	African American	1a	−731	342,000	
					−140	N/D	
					46	>700,000	Tacrolimus/Prednisone
					438	1,970	Tacrolimus
					1022	N/D	Prograf
I	59	Male	Caucasian	1a	N/A	433,210	N/A
J	60	Female	Caucasian	1a	N/A	638,441	N/A
K	53	Male	Caucasian	1a	−509	N/D	
					−47	N/D	
					58	352,000	Tacrolimus
					432	278,000	Tacrolimus/Prednisone
L	45	Male	African American	1a	N/A	523,391	N/A
M	65	Male	Caucasian	1a	−703	108,000	
					−33	N/D	
					48	2,570,000	Prograf
					395	3,540,000	Prograf
					696	4,890,000	Prograf
N	53	Male	Caucasian	1a	−602	369,000	
					69	1,710,000	Prograf
					195	1,670,000	Prograf
O	54	Female	Caucasian	1b	N/A	2,433,800	N/A
P	Unk	Unk	Unk	1b	N/A	2,841,586	N/A
Q	53	Male	Caucasian	1b	−1713	584,000	
					−311	N/D	
					44	N/D	Prograf/Prednisone
					229	N/D	Prograf
R	51	Male	Caucasian	1b	−715	47,100	
					−232	N/D	
					46	542,000	Prograf
					377	691,000	
					602	N/D	
S	74	Male	Caucasian	1b	N/A	523,530	N/A
T	57	Male	Caucasian	1b	−571	374,000	
					−24	N/D	
					29	687,000	Prograf
					365	9,830	Prograf

Time points indicate the number of days relative to the time of liver transplantation (number of days prior to liver transplant are indicated by a minus sign). For non-transplant patients with chronic HCV, time points and immunosuppression regimen are not applicable (N/A). N/D: not determined.

### Acquisition and analysis of pyrosequencing data

HCV RNA was amplified using primers complementary to HCV sequences. Primers were designed based on an alignment of 374 full-length HCV genotype 1 sequences from the Los Alamos HCV database [24]. All amplicons were sequenced bi-directionally. To minimize re-sampling of low viral load RNA templates, all samples were quantified by quantitative RT-PCR and a median 2.5×10^5^ copies of RNA per reaction were used in the amplification step.

After quality control, a total of 336,934 pyrosequence reads (3,633±1,538 reads per sample) were available for analysis. To distinguish authentic variants at drug resistance sites from technical artifacts, we subjected *in vitro* NS3 transcripts from a cloned H77C plasmid [25] to identical procedures including RT-PCR and pyrosequencing as patient-derived viral RNA. This provided a direct measure of technical error rates from RT-PCR and the sequence determination steps. Consistent with the published data [20], the overall mean error rate was ∼0.5%, with indels accounting for the majority of the errors (∼75% indels, ∼25% substitution errors). As reported previously [19], the mismatch error rate was position-dependent. Thus, we used position-specific background error rates to define authentic drug resistance mutations.

### Naturally occurring NS3/4A RAVs in chronic HCV

**Figure 2:**
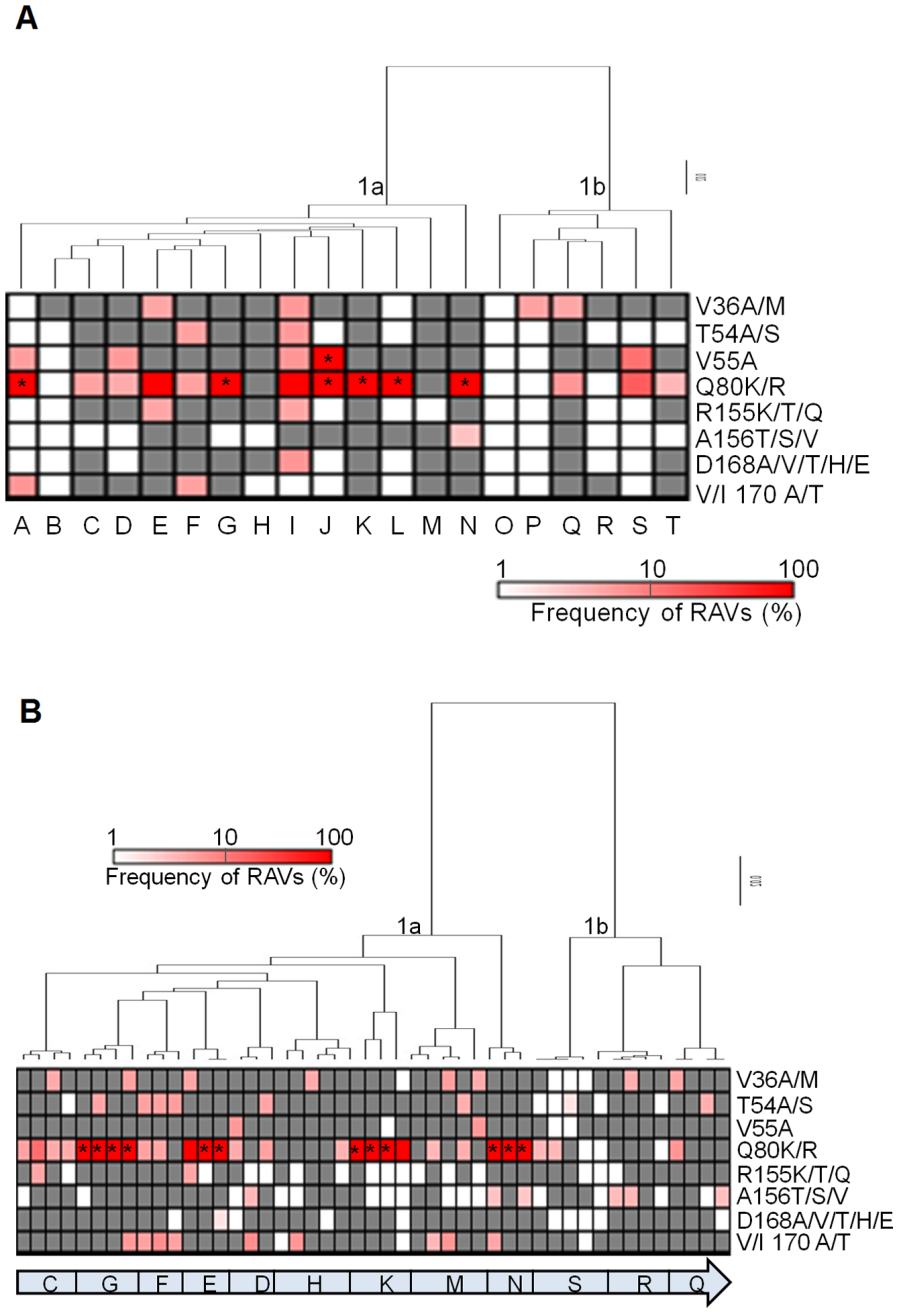
Frequency of NS3 resistance-associated variants (RAVs) in (A) chronic HCV and (B) longitudinal liver transplant recipients as determined by Roche/454 pyrosequencing. The proportion of pyrosequencing reads that harbored authentic RAVs is indicated by the intensity of magenta shown in the heatmap (p≤0.05, chi-square test). RAVs detected in pyrosequence reads that were not significantly enriched compared to background technical error rates are shown in grey (p>0.05, chi-square test). The background error rate was determined using *in vitro* control transcripts of known sequence that were amplified and sequenced in parallel with the RNA extracted from HCV subjects. Each column is a different sample, and each row represents a different RAV detected by pyrosequencing. Asterisks denote RAVs detected by direct Sanger sequencing. In (A), each column is a different subject (14 genotype 1a and 6 genotype 1b), and in (B), longitudinal samples within each subject (as indicated by the subject code at the bottom) are ordered from left to right, and phylogenetic analysis demonstrates that temporally associated samples are more closely related within subjects than samples between subjects.

NS3/4A inhibitors fall into two structural classes: linear ketoamides and macrocyclic compounds. Differences in their chemical scaffold have led to two major drug resistance profiles [26], [27]. For example, V36A/M, T54A/S, and V170A/T confer resistance to most linear ketoamides, whereas variants at Q80 and D168 are associated with resistance to macrocyclic compounds. Substitutions at R155K/T and A156V/T/S confer cross-resistance between the two classes [26]. To assess the abundance of RAVs in chronic HCV infection, we used pyrosequencing to analyze 20 HCV quasispecies including 8 samples from chronic HCV and 12 pre-LT samples from LT recipients. We queried eight main amino acid positions known to be associated with NS3/4A resistance (V36, T54, V55, Q80, R155, A156, D168, and V/I170) (Figure 2A). We considered drug resistance calls as authentic if the frequencies determined by pyrosequencing reads were significantly enriched compared to the background technical error rates (p<0.05; chi-square). Overall, 31 of the 104 RAVs detected in our pyrosequence reads (29.8%) were considered authentic RAVs.

As expected, conventional sequencing detected the most abundant RAVs (Figure 2, asterisks), while clonal sequencing detected additional minor variants (Supporting Information S1). Fourteen of 20 subjects (70%) harbored no dominant RAVs as determined by population sequencing. No dominant RAVs were observed at positions 36, 54, 155, 156, 168 and 170, consistent with observed low prevalence of high-level, naturally occurring PI-resistant variants reported previously [12], [28]. Overall, conventional sequencing failed to identify 77.4% (24 of 31) of authentic PI-resistant substitutions, most of which were low-frequency variants. These results demonstrate that naturally occurring RAVs are common but most RAVs circulate at low frequencies not readily detectable by conventional sequencing.

### Temporal dynamics of HCV quasispecies in liver transplantation

To examine the evolution of HCV quasispecies, we analyzed 1–2 samples pre-LT and 2–3 samples post-LT for 12 liver transplant recipients (9 GT1a and 3 GT1b). Phylogenetic analysis based on the pyrosequencing reads revealed that temporally associated intra-host HCV quasispecies populations were more closely related to one another than HCV populations between subjects (Figure 2B). To investigate intra-host HCV evolution, we clustered all pyrosequence reads into operational taxonomic units (OTUs) at 97% sequence identity, which allowed us to track changes in major intra-host HCV variants over time.

**Figure 3:**
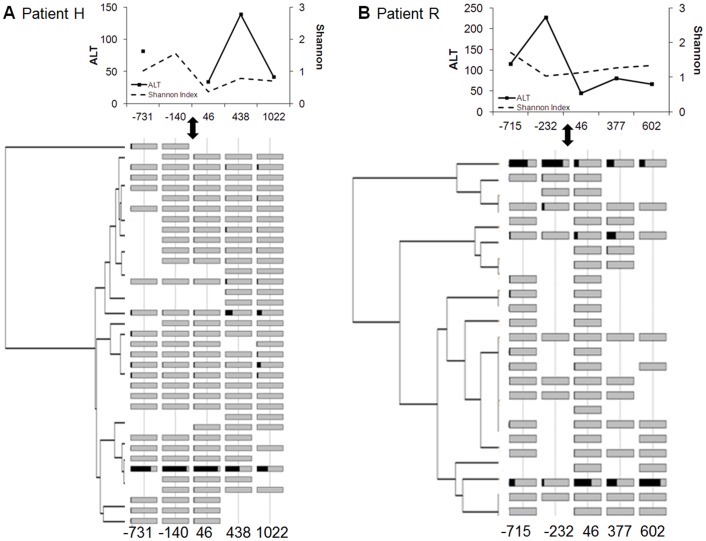
Dynamics of major NS3 variants in liver transplant recipients. Forward pyrosequence reads (∼337 nt, corresponding to coordinates 3342 to 3674 on the H77 genome, accession NC_004102) were clustered into operational taxonomic units (OTUs) at 97% sequence identity. The relative abundance of each major variant is shown by the black shading, where the extent of the black region from left to right within the gray bar indicates the proportion in the total viral population. The black double-headed arrow denotes the time of liver transplantation, and the number of days before and after liver transplantation are indicated at the bottom. Trees were generated using UPGMA. Shannon diversity values based on NS3 pyrosequence reads represent changes in NS3 diversity over time (Top panels). ALT: alanine aminotransferase. (A) The major variant that established re-infection post-LT was identical or closely related to the dominant variant pre-LT (B) Two minor variants pre-LT became dominant quasispecies post-LT.

In all LT subjects analyzed, 1–3 major variants dominated the viral populations. Two major patterns were evident (Figure 3). In 9 subjects, the major variants that established re-infection post-LT were identical or closely related to the dominant variants pre-LT (Figure 3A). For the remaining 3 subjects, 1–2 minor variants pre-LT became dominant post-LT (Figure 3B; data for all subjects are shown in Supporting Information S1). In many subjects, the overall genetic diversity immediately post-LT was low compared to pre-LT (Shannon Index; Figure 3A and Supporting Information S1). Despite the restricted diversity post-LT, all major variants post-LT share a common ancestor with pre-LT lineages, and no major clades were extinguished following LT.

We next asked whether the frequency and stability of variants associated with NS3 resistance were influenced by the temporal changes in intra-host HCV variants. In most subjects, the abundance of RAVs was variable over time (Figure 2B). Notably, Q80K was a common variant, detected in 28 of 47 samples. Variants at positions V36, T54 and V55 were detected in 13% of the samples, whereas substitutions at positions 155, 156 and 168 were uncommon (only 6.4% of the samples). With the exception of Q80K, variants associated with NS3 resistance were minor variants in nearly all cases.

### Linkage mapping of drug resistance mutations

Linkage of mutations at V36 or T54 with R155 or A156 is commonly selected in patients failing PI monotherapy and is known to confer intermediate to high-level PI resistance [17], [29]. Thus, it was of great interest to develop novel sensitive approaches for mutational linkage analysis *in vivo*. Current sequencing approaches suffer from several limitations. Population sequencing cannot conclusively demonstrate linkage between different substitutions on the same viral genome. Determining linkage of genetic variants at low frequencies using the clonal sequencing approach could be labor-intensive. For our pyrosequencing datasets, the forward and reverse pyrosequencing reads (after trimming barcode and primer sequences) did not have sufficient overlaps to allow reconstruction of haplotypes for linkage analysis. Because the Illumina paired-end sequencing technology can read both the forward and reverse strands of each amplicon to provide long range sequencing information during one paired-end read, we asked whether the paired-end approach was suitable for long-range linkage analysis in HCV.

The Illumina platform uses dye-terminated primer extension to sequence DNA. The algorithm for base calling relies on fluorescent intensities from the first several nucleotides incorporated to normalize the fluorescent signals for subsequent nucleotide extension. Thus, we first engineered barcodes that varied between 4 and 8 nucleotides in length to reduce the likelihood that adjacent clusters on the Illumina solid support would be scored as one amplicon during sequencing. Next, we chose barcode sequences to ensure that at least three different nucleotides were represented. Lastly, we modified procedure for library preparation to accommodate the standard paired-end sequencing protocol on the Illumina platform (Figure 1).

We first introduced double mutations (T54A/R155K) using a plasmid containing the wild-type H77C sequence and confirmed the mutations by Sanger sequencing. Next, we synthesized their transcripts *in vitro*, then reverse-transcribed and paired-end sequenced the RT-PCR products. The sequencing data confirmed that amino acid substitutions at position T54 and R155 for the T54A/R155K double mutant were linked in >99.6% of the paired-end reads (Table 2, T54A+R155K). Based on the WT data, the background technical error rate was determined to be <0.2% (Table 2, WT; 321,303±202,093 reads per sample). Next, we constructed four mock communities of *in vitro* RNA transcripts to determine whether the mutation linkage in T54A+R155K could be detected at low frequencies (Table 3). As shown in Population 2, double mutant variants in as low as 0.5% of the total RNA pool could be detected. The low prevalence and low abundance of naturally occurring R155 and/or A156 mutations precluded detailed analysis of low frequency linkage for most of the samples in our cohort. Nevertheless, we chose 5 clinical samples (Table 2, Clinical Samples) in which the R155 and/or A156 mutations were detected at low frequencies by pyrosequencing (although not statistically enriched over the background error rate) for linkage analysis. Double mutant variants associated with high-level PI resistance were not detected in any of the five clinical samples (i.e. all were below the background error rate). Overall, mutation frequencies were in good agreement between the Roche/454 and Illumina data sets (Supporting Information S1).

**Table 2:** Mutation linkage analyzed by Illumina paired-end sequencing.

	V36	T54	V55	R155	A156	D168	I170	V36/R155	V36/A156	T54/R155	T54/A156
***Control***											
WT	0.1	0.1	0.17	0.025	0.07	0.07	0.04	0	0	0.0007	0.00035
T54A	0.02	96.2	0.003	0.0003	0.42	0.0015	0.0005	0.00019	0.01	0.034	1.56
T54A+R155K	0.0005	0.3	0.0005	0.1	0.0001	0	0	0.128	0	99.6	0.05
***Clinical Samples***											
E-1	0.29	0.3	0.34	0.09	0.11	0.16	0.27	0	0	0	0
G-4	0.19	0.15	0.29	0.03	0.09	0.06	0.14	0	0	0.001	0
I	0.19	0.22	0.29	0.05	0.094	0.17	0.16	0	0.0027	0.003	0.0003
J	0.001	0.004	0.98	0.0003	0	0	0.0003	0.0003	0.0003	0.0007	0.0003
Q-1	0.15	0.21	0.67	0.019	0.07	0.13	0.14	0	0	0	0.0009

Each row corresponds to an individual sample (control transcripts or clinical samples).

Each column corresponds to single or double mutant variants associated with PI resistance (WT codons are listed), and their mutation frequency (defined as non-WT) is shown (%). Technical error rate was determined as 0.2% based on the WT control data.

**Table 3:** Illumina paired-end sequencing identifies low frequency T54A+R155K double mutant in mock RNA populations.

	Population 1		Population 2		Population 3		Population 4	
	*Expected*	*Observed*	*Expected*	*Observed*	*Expected*	*Observed*	*Expected*	*Observed*
WT	92.6	92.77	87.1	91.16	98.6	96.37	35.2	48.6
T54A	1.3	6.0	12.3	8.28	1.2	3.5	44.5	42.76
T54A/R155K	6.0	1.08	0.56	0.45	0.058	0.029	20.4	8.07

RNA transcripts were synthesized from WT, T54A, or T54A+R155K plasmid *in vitro*. Each mock population of RNA transcripts was constructed according to the proportions indicated in the “Expected” column. The proportion of paired-end reads that harbored T54A single mutant, T54A/R155K double mutant, or WT are shown in the “observed” column (%). While the double mutant at a level below 0.1% was detected (Population 3), it was below our experimentally determined threshold for background error rate of ∼0.2% (see Table 2).

## Discussion

This study presents an in-depth analysis of NS3 quasispecies and resistance-associated variants (RAVs) in chronic HCV infection and liver transplantation. With NS3/4A protease inhibitors now in the clinic, there is a great deal of enthusiasm for HCV therapeutics. While peginterferon-ribavirin still remains the backbone of antiviral therapy, an interferon-free regimen with combination DAAs will likely become a reality [9] and drug resistance may be an important consideration in the foreseeable future. Here, we used Roche/454 pyrosequencing to determine abundance and *Illumina* paired-end sequencing to quantify mutation linkage of naturally occurring RAVs in chronic HCV. In addition, we describe temporal changes in viral populations during liver transplantation.

Naturally occurring drug resistance mutations preexist in treatment-naïve individuals [12]. A recent survey using population sequencing suggests that the prevalence of NS3 RAVs is low in genotype 1 DAA-naïve patients [30]. However, since population sequencing reports only the most predominant nucleotides in a given sequence, the conventional method is not sensitive in detecting minor variants present in less than 20% of the viral population. Thus, the baseline prevalence of NS3 RAVs may be higher if more sensitive techniques were used. Importantly, linkage of minor variants from different parts of the genome is not possible with population sequencing. In our cohort, conventional sequencing failed to detect minority variants in most subjects, while some RAVs could be identified using more extensive clonal sequencing. Our data demonstrate that naturally occurring RAVs are common in transplant recipients both before and after LT. The prevalence of Q80K/R polymorphism was particularly high, approaching 70–80% in our cohort (78% of genotype 1a and 50% of genotype 1b samples), which were significantly higher than previously reported [30], [31]. Interestingly, Q80K was frequently a dominant variant in the viral swarm, but substitutions at V36, T54 and V55 were generally minor variants (<5% of pyrosequence reads). Variants conferring high-level drug resistance (i.e. substitutions at 155, 156 and 168) were rare; no dominant mutation at these positions was detected in our dataset. These data are consistent with the recent data obtained using population sequencing methods [30], in which HCV variants with lower-level resistance were detectable but higher-level resistant variants were not observed. Our data is also consistent with the hypothesis that the abundance of RAVs may be a function of viral fitness in quasispecies populations. In a recent study, RAVs were detected in most subjects who failed to achieve SVR following combination therapy that includes protease inhibitor. Following the withdrawal of DAAs, different RAVs were gradually replaced by wild-type virus at different rates over time, presumably due to the differential fitness of the specific resistant variants [32], [33].

A significant advance of this study is the novel application of the *Illumina* paired-end sequencing technology to mutation linkage analysis. To our knowledge, this is the first report of mapping linkage of viral drug resistance using this approach. Using *in vitro* control transcripts, we demonstrate that linked variants constituting as low as 0.5% of the overall RNA population could be detected and their linkage confirmed. The differences between the predicted and measured frequencies in our mock populations could be explained by differential PCR amplifications leading to skewing of allelic frequencies. In HIV, it has been shown that PCR amplification could skew the abundance measurement by 2 to 15 fold, and in some cases the effects can be severe (up to 100-fold) [34]. Going forward, it would be of interest to improve upon our current methods by combining strategies such as PrimerID [34] with the sensitive Illumina approach employed in this study. The combined approach could control for allelic skewing as well as template resampling and sequencing errors, thereby allowing for more accurate quantification of mutation linkage. In this study, double PI mutants were not detected in our PI-naïve cohort. This finding was not surprising as double PI mutants likely suffer from poor fitness compared to WT and the frequency of resistance variants is generally determined by its replicative fitness. Since mutations at V36 or T54 linked to R155 or A156 confer high-level resistance to NS3/4A inhibitors, the paired-end sequencing approach should be suitable for sensitive NS3 linkage analysis during pretreatment and early DAA therapy. More broadly, the paired-end strategy could be applied to other drug-targeted loci in HCV as well as other viruses such as HIV and HBV.

A major challenge in studies of viral quasispecies and RAVs using next-generation sequencing technology is the potential erroneous drug resistance calls due to technical artifacts. These artifacts could arise during sample preparation, including reverse transcription and PCR, as well as from the Roche/454 or Illumina sequence determination step. We took several quality control measures to estimate and correct for the background technical error rates. First, we quantified RNA templates by quantitative RT-PCR to minimize resampling of low viral load RNA templates (our one-step RT-PCR procedure did not permit direct measurement of cDNA copy numbers), and we observed no correlations between input RNA template and quasispecies diversity to suggest template resampling (Supporting Information S1). Secondly, we used *in vitro* transcribed RNA rather than plasmid DNA as controls to correct for errors introduced during the RT step. Although errors generated during *in vitro* transcription could artificially inflate the measured error rate, RNA polymerase error rate is generally lower compared to that of RT which has been previously shown to be a negligible source of errors compared to pyrosequencing errors [19]. Next, through a combination of global and pairwise sequence alignments followed by manual inspection of reads containing codon-changing nucleotide substitutions, we conservatively called authentic RAVs only if the detected variant was statistically enriched over position-specific errors determined for the control RNA transcript. The technical error rates determined in our control experiments were consistent with error rates published previously [21] [35], [36]. Thus, while our 454 dataset revealed a large number of RAVs (i.e. grey and magenta boxes in Figures 2 and 3), only ∼30% of the detected RAVs were considered authentic by our stringent criteria.

Longitudinal analysis of LT recipients allowed us to track temporal changes of intra-host viral variants. In most subjects, the predominant viral variant remained relatively stable over several years, whereas in others, a minor variant pre-LT became a major variant post-LT. In all subjects, the major viral variants post-LT shared a common ancestor with viral lineages pre-LT. These results are consistent with the recent clonal sequencing data based on hypervariable envelope sequences in the same transplant cohort [37], but differ from an earlier study using clonal analysis, where viral genetic bottleneck was observed for the short period immediately following liver transplantation (<1 months) [38]. It would be of great interest to confirm the stability of HCV populations and RAVs in other LT cohorts, as well as other longitudinal cohorts such as treatment-naïve chronic HCV and HIV-HCV co-infection, as this will have a direct impact on the selection of effective DAA agents for combination antiviral therapy.

Intense efforts are presently focused on the development of therapeutics for HCV. Our results indicate that RAVs pre-exist in the viral swarm at low levels not readily detectable by conventional sequencing in most patients. This study also demonstrates that mutation linkage at low levels could be detected using our modified *Illumina* paired-end sequencing approach. Although recent data suggest that the presence of DAA-resistant variants in treatment-naïve patients at baseline receiving IFN and protease inhibitor- containing combination therapy does not impact sustained viral response rate, the effect of specific resistant variant frequency on the response to IFN-free DAA regimens remains clear. As we move toward an era of interferon-free oral antiviral therapy, the determination of pre-existing RAVs prior to therapy may be important as nearly 100% of new livers are re-infected by viruses present in the bloodstream, old liver or peripheral blood mononuclear cells [1], [39], [40]. The full clinical significance of these pre-existing RAVs remains to be defined in prospective studies, but is important as interferon-based therapy is currently the only approved treatment for post-LT HCV re-infection. The methods described here should be widely applicable to detailed studies of pre-existing and low-level drug resistance, mutation linkage, and viral dynamics in patients undergoing direct acting antiviral therapies.

## Supporting Information

Supporting Information S1 **Combined Supporting Information file containing supplementary methods, tables and figures.** Supplementary methods include information on RNA isolation, primer design/RT-PCR amplification, RNA transcript/mock community construction, population/clonal sequencing, pyrosequencing platforms, and bioinformatics processing. Supplementary tables include information on primers, read filtering, variant frequencies, and alpha diversity. The supplementary figure covers quasispecies evolution and dynamics in liver transplant recipients.
(PDF)
