# Supplementary material for: Deep Sequencing Analysis of HCV NS3 Resistance-Associated Variants and Mutation Linkage in Liver Transplant Recipients
Source: PLoS One. 2013 Jul 29;8(7):e69698. doi: 10.1371/journal.pone.0069698 (PMC3726766; doi:10.1371/journal.pone.0069698)
Supplement: Supporting Information S1 — Combined Supporting Information file containing supplementary methods, tables and figures. Supplementary methods include information on RNA isolation, primer design/RT-PCR amplification, RNA transcript/mock community construction, population/clonal sequencing, pyrosequencing platforms, and bioinformatics processing. Supplementary tables include information on primers, read filtering, variant frequencies, and alpha diversity. The supplementary figure covers quasispecies evolution and dynamics in liver transplant recipients. (PDF) [file pone.0069698.s001.pdf]

Supplementary Information for *Kirst et al.*

Supplementary Methods

HCV RNA isolation: Viral RNA was extracted from plasma samples using the QIAamp Viral RNA Mini kit (Qiagen, Valencia, CA). HCV RNA was quantified by quantitative RT-PCR using primers specific to the highly conserved 5' UTR of the HCV genome using the Rotor-Gene SYBR green RT-PCR kit (Qiagen, Valencia, CA).

Primer design and RT-PCR amplification: Gene specific RT-PCR primers were designed based on an alignment of 374 full-length HCV genotype 1 sequences from the Los Alamos HCV database (http://www.hcv.lanl.gov, accessed on 7/7/2009). Roche/454 primers were composites containing the required sequences for titanium chemistry procedure, a unique 8-base DNA barcode that indexed each sample, and HCV-specific primer sequences F3342 and R3973 (Supplementary Table S1). Primers for Illumina paired-end sequencing were composites of partial PE sequence PE1 and PE2, a unique DNA barcode (4-8 bp) and HCV-specific primer sequences F3507 and R3973 (Supplementary Table S1). All samples were amplified using SuperScript III one-step RT-PCR Platinum Taq HiFi (Invitrogen, Carlsbad, CA) following manufacturer's recommendations. This kit was chosen to minimize nucleotide misincorporation errors during PCR. The reverse transcription (RT)-PCR cycling conditions were as follows: 1 cycle at 50°C for 60 min, followed by 15 min at 95°C;

30 or 35 cycles of denaturation at 94°C for 30 s, annealing at 48°C for 30 s, and then extension at 72°C for 1 min; and final extension at 72°C for 10 min. Samples with a viral load of greater than 5x 10^7 copies/mL were amplified for 30 cycles, whereas 35 cycles were used for samples with lower viral loads.

To estimate the technical error rates for our procedure, control RNA of known sequence was generated by *in vitro* transcription of linearized plasmid containing a T7 promoter and full-length H77C genotype 1a sequence using MEGAscript T7 kit (Ambion, Austin, TX). Prior to *in vitro* transcription, the plasmid was linearized using EcoRI (Promega, Madison, WI) that cuts downstream of HCV NS3. Control RNA transcripts were subjected to identical experimental procedures including RT-PCR and pyrosequencing as patient-derived viral RNA.

Construction of NS3 in vitro RNA transcripts and mock communities: Site-directed mutagenesis at amino acid positions 54 and 155 of the NS3 gene was performed using the H77C genome as the template and the Quickchange site directed mutagenesis kit, following manufacturer's instructions (Agilent, Santa Clara, CA). Plasmids containing the mutations were linearized and transcribed *in vitro* as described above. Mock RNA communities were constructed using different concentrations of wild-type, single (T54A) and double (T54A/R155K) mutant transcripts, and were subjected to identical amplification and deep sequencing procedures as the extracted RNA from clinical specimens.

Population and clonal sequencing: Direct sequencing of the NS3 gene was performed using primers (2943F and 4231R, Supplementary Table S1) upstream and downstream from the Roche/454 and Illumina primer binding sites. This allowed determination of both the NS3 consensus, which specifies the most predominant nucleotide at each position, and the sequences at the primer binding sites. For clonal sequencing, RT-PCR products amplified using 454 fusion primers were cloned into pCR2-TOPO vectors and transformed into chemically competent TOP10 cells (Invitrogen, Carlsbad, CA). Up to 32 clones per sample were sequenced. Phylogenetic analysis based on the population sequencing data was performed using Geneious and the Phangorn phylogenetic analysis package in R.

Roche/454 pyrosequencing: RT-PCR products amplified using 454 fusion primers were separated by agarose gel electrophoresis, and the fragments of expected size excised and extracted using the Qiagen gel extraction kit (Qiagen, Valencia, CA). Gel-purified PCR products were quantified using Qubit (Invitrogen, Carlsbad, CA), pooled by equimolar concentrations, and subjected to bidirectional pyrosequencing using the titanium chemistry.

***Illumina paired-end sequencing**:* The Illumina platform uses dye-terminated primer extension to sequence DNA. The algorithm for base calling relies on fluorescent intensities from the first several nucleotides incorporated to normalize the fluorescent signals for subsequent nucleotide extension. To reduce the likelihood that adjacent clusters on the Illumina solid support would be scored as one amplicon during sequencing, we engineered barcodes that varied between 4 and 8

nucleotides in length. In addition, we chose barcode sequences to ensure that at least three different nucleotides are represented. Amplified NS3 gene segments (from 5 clinical samples and 2 control *in vitro* transcripts, Table 2) were purified from electrophoresis gel slices, quantified using Qubit (Qiagen, Valencia, CA) and pooled at equimolar amounts. Purified amplicons were then tailed with flow cell adaptors (12 cycles of PCR amplification, Figure 1). The prepared library was quantified using Kapa Library Quantification kit (Kapa Biosystems, Woburn, MA) and sequenced on Illumina Genome Analyzer IIx at the University of Florida ICBR sequencing core.

Bioinformatic analysis: Pyrosequence reads were processed using the following quality control criteria: (i) an exact match to the barcode and the primer sequences, (ii) > 360 bases for forward reads; > 290 bases for reverse reads in length before trimming the barcode and primer sequences, and (iii) no ambiguous bases (Ns). Reads were grouped based on barcodes, then barcodes and primer sequences were trimmed. Each read was further trimmed to a final length of ~337 nucleotides for forward reads and ~264 nucleotides for reverse reads. The trimmed reads were aligned to the H77C reference sequence using global multiple sequence alignment (POA - “Partial Order Alignment” multiple sequence aligner) (1), then the codon positions associated with resistance to protease inhibitors were identified (custom R scripts were created) (2). Of the reads that contain codon-changing nucleotide substitutions, pairwise sequence alignments (ClustalW2) (3)were performed followed by manual inspection of the aligned sequences. This second quality control step ensured that the observed nucleotide substitutions did not arise from sequence alignment errors. Clustering and OTU

formation were carried out using the USEARCH/UCLUST suite (4). Phylogenetic analysis was performed using UPGMA based on the population sequencing data and the Phangorn phylogenetic analysis package in R (2).

To estimate the technical error rates, PCR products amplified from *in vitro* transcripts of known NS3 sequences (as described above) were subjected to pyrosequencing. The overall mean error rate including insertions, deletions and substitutions was ~0.5%. To distinguish authentic variants at drug resistance sites from technical artifacts due to nucleotide misincorporation during PCR amplification and pyrosequencing, position-specific background error rate was calculated to define authentic drug resistance mutations using a chi-square test.

Illumina paired-end reads were processed using the following criteria: (i) exact match to barcode and primer sequences; (ii) no ambiguous bases; (iii) minimum length of 75 bases after trimming so it would cover amino acid positions V36 through V55 in forward amplicon (PE1), and R155 through I170 in reversed amplicon (PE2) and (iv) both reverse and forward sequences passed all previous quality steps (no reads with unknown ‘B’ quality scores and no reads that failed Illumina quality check ‘0’). The filtered, trimmed reads were then aligned to H77C reference sequence, and the codon positions associated with PI resistance were identified and the frequency of resistance mutations calculated (custom R scripts were created).

References

1. Lee C, Grasso C, Sharlow MF. Multiple sequence alignment using partial order graphs. Bioinformatics 2002; **18**(3): 452-464.

2. Schliep KP. Phangorn: Phylogenetic analysis in R. Bioinformatics 2011; **27**(4): 592-593.

3. Larkin MA, Blackshields G, Brown NP, Chenna R, McGettigan PA, McWilliam H, Valentin F, et al. Clustal W and clustal X version 2.0. Bioinformatics 2007; **23**(21): 2947-2948.

4. Edgar RC. Search and clustering orders of magnitude faster than BLAST. Bioinformatics 2010; **26**(19): 2460-2461.

Supplementary Table S1: Primers used for PCR amplification of HCV followed by 454 pyrosequencing or Illumina paired-end sequencing. H77C genome coordinates for each forward and reverse primer is shown in the numerical portion of the primers name. “xxx” denotes the location of unique barcode sequences (4 or 8 bp).

**Primer ID**	**5’-> 3’ sequence**
454-A-BC-3342F (forward)	CGTATCGCCTCCCTCGCGCCATCAGAGxxxxxxxxCCYGTYTCYGCYCGNAGRGG
454-B-BC-3973R (reverse)	CTATGCGCCTTGCCAGCCCGCTCAGxxxxxxxxGTRAANACCGGRGAYCKCATRGT
NS3 gene specific 2943F (Forward)	GGRGGSCGCGAYGCCRTCATC
NS3 gene specific 4231R (reverse)	CCGTAGGTGGARTAYGTGAT
Illumina-PE1-3507F (forward)	ACACTCTTTCCCTACACGACGCTCTTCCGATCTxxxxGAGGGTGAGGTYCAGAT
Illumina-PE2-3973R (reverse)	CGGCATTCCTGCTGAACCGCTCTTCCGATCTxxxxGTRAANACCGGRGAYCKCAT
PE1	AATGATACGGCGACCACCGAGATCTACACTCTTTCCCTACACGACGCTCTTCCGATCT
PE2	CAAGCAGAAGACGGCATACGAGATCGGTCTCGGCATTCCTGCTGAACCGCTCTTCCGATCT

Supplementary Table S2: Filtered sequence read attrition tables. The tables are divided by the sequencing platform(454 bi-directional or Illumina paired-end). Only the final number of reads that passed quality criteria checks was used in subsequent analyses. Manual rejection of additional sequences was performed in some cases. There were different minimum length requirements for each end of the 454 reads, depending on the amino acid position coverage needed. The Illumina reads utilized a unified barcode scheme for pairing the read ends; the final read counts on both ends correspond with each other.

454 Bi-directional sequencing

Cross-Sectional

Subject	# of Reads Matching Barcode & Primer	# of Reads With No Ambiguous Base Calls	# of Reads With Minimum Length (A forward end)
A	3749	3473	3460
B	4000	3342	3332
I	5438	4508	4490
J	3179	2809	2807
L	3706	2859	2854
O	4046	3948	3947
P	4873	4803	4788
S	3614	3292	3288

Subject	# of Reads Matching Barcode & Primer	# of Reads With No Ambiguous Base Calls	# of Reads With Minimum Length (B reverse end)
A	2526	2496	2496

B	3109	2988	2988
I	4065	3848	3848
J	2058	1972	1972
L	2600	2537	2535
O	2749	2098	2098
P	3466	3417	3413
S	2907	2457	2455

Longitudinal

Subject	# of Reads Matching Barcode & Primer	# of Reads With No Ambiguous Base Calls	# of Reads With Minimum Length (A forward end)
C-1	9057	8114	4534
C-2	7836	7072	3842
C-3	9811	8907	5057
C-4	7297	6678	3607
D-2	6279	5837	3624
D-4	6238	5910	4023
D-5	5025	4796	2650
E-2	10408	9369	5390
E-3	9687	7761	4585
E-4	10894	9972	6328
F-1	9304	6309	3445
F-3	7405	5215	2666
F-4	8346	5326	2869
G-1	7755	6485	4093
G-2	9434	7322	4052
G-4	10471	7687	4364
G-5	12041	9302	5067
H-1	6987	6150	4389
H-2	4654	4075	2563

H-3	9862	8833	6017
H-4	6958	6703	4524
H-5	7631	7326	4852
K-1	8682	8224	5814
K-2	9847	9300	6365
K-3	3208	3020	1976
K-4	5429	5139	3127
M-1	8907	8033	4525
M-2	10116	9352	6099
M-3	11064	10125	5704
M-4	7483	6823	4271
M-5	8992	8357	5635
N-1	9623	6868	3127
N-3	15701	8576	3793
N-4	12842	7435	1940
Q-1	9481	8342	3558
Q-2	10395	9147	3823
Q-3	10334	9605	4681
Q-4	9600	8849	3929
R-1	8558	7709	4929
R-2	8827	7531	5758
R-3	13768	11712	8690
R-4	10881	9285	6901
R-5	6997	5968	4422
T-1	10005	8267	5464
T-2	8040	6637	4439
T-3	9454	7868	5266

**Subject**	**# of Reads Matching Barcode & Primer**	**# of Reads With No Ambiguous Base Calls**	**# of Reads With Minimum Length (B reverse end)**
C-1	8745	8198	5221

C-2	6699	6320	3797
C-3	9701	8959	5742
C-4	7018	6588	4094
D-2	6174	5548	3600
D-4	5922	5545	3183
D-5	5321	4877	2784
E-2	10090	9365	5909
E-3	9564	8968	5966
E-4	10541	9929	6493
F-1	8948	8496	5578
F-3	7034	6764	4221
F-4	8815	8411	5304
G-1	7176	6440	4006
G-2	9980	9228	6117
G-4	9859	9442	6450
G-5	11299	10651	6567
H-1	6434	5403	3160
H-2	4789	4394	2581
H-3	9529	8239	5142
H-4	7113	6545	3729
H-5	6154	5666	3135
K-1	8089	7501	4701
K-2	9719	8221	5128
K-3	3570	3160	1987
K-4	5165	4554	2402
M-1	8291	8010	5051
M-2	9918	9495	6364
M-3	10534	10152	6658
M-4	5525	5321	3512
M-5	8600	8234	5652
N-1	9338	8749	4925

N-3	14966	14140	8217
N-4	14850	13944	8035
Q-1	8354	7562	4677
Q-2	9606	8764	5380
Q-3	9597	8557	5263
Q-4	8622	7712	4663
R-1	8103	7428	5379
R-2	8448	7105	4552
R-3	13136	11381	6894
R-4	10871	9393	5798
R-5	6493	5892	3426
T-1	9057	7989	5382
T-2	7766	6934	4409
T-3	8757	7849	4951

Illumina paired-end sequencing

Paired End 1 - Forward

Subject	# of Reads Matching barcode	# of Reads Matching Barcode & Primer	Linked
E-1	155926	148747	105034
G-4	132011	126573	89117
I	387232	368768	294835
J	358224	343425	275752
Q-1	162741	155886	116018
Mock population 1	80810	78023	60663
Mock population 2	598568	579598	461144
Mock population 3	730781	706266	581334
Mock population 4	717199	696689	562961

Paired End 2 - Reverse

Subject	# of Reads Matching barcode	# of Reads Matching Barcode & Primer	Linked
E-1	152428	142381	105034
G-4	128104	119549	89117
I	392692	366546	294835
J	359227	335691	275752
Q-1	161999	150590	116018
Mock population 1	107161	102835	60663
Mock population 2	586872	557877	461144
Mock population 3	731147	705075	581334
Mock population 4	720382	689124	562961

Supplementary Table S3: Resistance-associated variants (RAVs) detected by clonal sequencing compared to population sequencing in selected samples. Eight amino acid positions known to be associated with NS3/4A resistance (V36, T54, V55, Q80, R155, A156, D168, and V/I170) were queried. For clonal sequencing, the denominator denotes the number of clones sequenced, and the numerator represents the number of clones with the indicated RAVs.

**Subject**	**Population Sequencing**	**Clonal sequencing**
A	V36L; Q80K	7/9 V36L; 6/9 Q80K
D-2	WT	3/15 V55A; 1/15 Q80K
F-3	WT	2/15 V55A; 3/15 Q80K
H-2	WT	5/26 V55A; 6/26 Q80K
I	Q80K	9/9 Q80K
L	V55A; Q80K	4/9 V55A; 4/9 Q80K
P	N/A	1/9 T54A

Supplementary Table S4: Comparison between RAVs frequency observed in clinical samples through 454 and illumina sequencing technologies.

	**V36**	**T54**	**V55**	**R155**	**A156**	**D168**	**I170**
Illumina sequencing							
E-2	0.29	0.3	0.34	0.09	0.11	0.16	0.27
G-4	0.19	0.22	0.29	0.03	0.09	0.06	0.14
I	0.19	0.15	0.29	0.05	0.09	0.17	0.16
J	0.001	0.004	92.8	0.0003	0	0	0.0003
Q-1	0.15	0.21	0.67	0.019	0.07	0.13	0.14
Roche 454 sequencing							
E-1	0.25	0.12	0.25	0.25	0.06	0.12	0.14
G-4	0.08	0.03	0.14	0.02	0.06	0.16	0.06
I	0.35	0.35	0.44	0.23	0.05	0.41	0
J	0.17	0	99.5	0	0.15	0	0
Q-1	0.23	0.13	0.19	0.03	0.06	0.09	0.03

Supplementary Table S5: Comparison of input RNA copy number, number of reads, number of OTUs, and Shannon diversity values for pyrosequenced longitudinal transplant samples.

Subject ID/timepoint	Input RNA copy # for RT-PCR	# of 454 seqs	# of OTUs	Shannon Index Value
C-1	10445	4337	26	1.728030698
C-2	4440	1860	26	1.792062014
C-3	223507.5	4992	25	0.721194117
C-4	228159	3510	15	0.814135817
D-2	5845	1469	25	1.913179586
D-4	101502.5	3897	25	1.166052594
D-5	237507.5	2587	25	1.44593846
E-2	1615	4701	49	2.886074557
E-3	910	4328	34	1.788510412
E-4	1500	6096	16	0.518154068
F-1	97030	2628	37	1.143161307
F-3	303453	2518	38	2.157644747
F-4	410006	2727	34	1.760810551
G-1	15385	810	20	1.906313941
G-2	10610	1757	24	1.463779243
G-4	22325	3459	24	1.908053919
G-5	42355	4902	23	1.383503286
H-1	87500	2356	20	1.135624485
H-2	53042.5	1130	10	0.835522806
H-3	172205	5801	25	1.329452261
H-4	122743	4459	14	1.285389902
H-5	379776	4808	12	0.619929629
K-1	110802.5	5736	13	1.061587413
K-2	35730	6338	15	0.542071981
K-3	9480	1961	7	0.274934318
K-4	15075	2934	8	0.602752516
M-1	9590	3986	65	2.681293378
M-2	83170	5913	69	2.24831665
M-3	49130	5528	74	2.458326425
M-4	149347.5	4205	57	2.137669522
M-5	69575	5521	45	1.072188133
N-1	1120	2256	11	1.285354518

N-3	29835	3724	5	0.413763641
N-4	36535	1841	8	0.568391725
Q-1	4810	3030	41	1.80878767
Q-2	9440	3448	42	1.86903577
Q-3	10465	4519	31	1.279304281
Q-4	4270	3358	21	0.76204842
R-1	3915	1017	22	1.125352602
R-2	120872.5	5655	32	0.535850953
R-3	24085	8508	32	0.563006298
R-4	223040	6803	32	1.536806081
R-5	208175	4347	30	1.966300207
T-1	9895	5167	2	0.126567195
T-2	18775	4404	3	0.214043522
T-3	17430	5188	3	0.169623003

Supplementary Figure

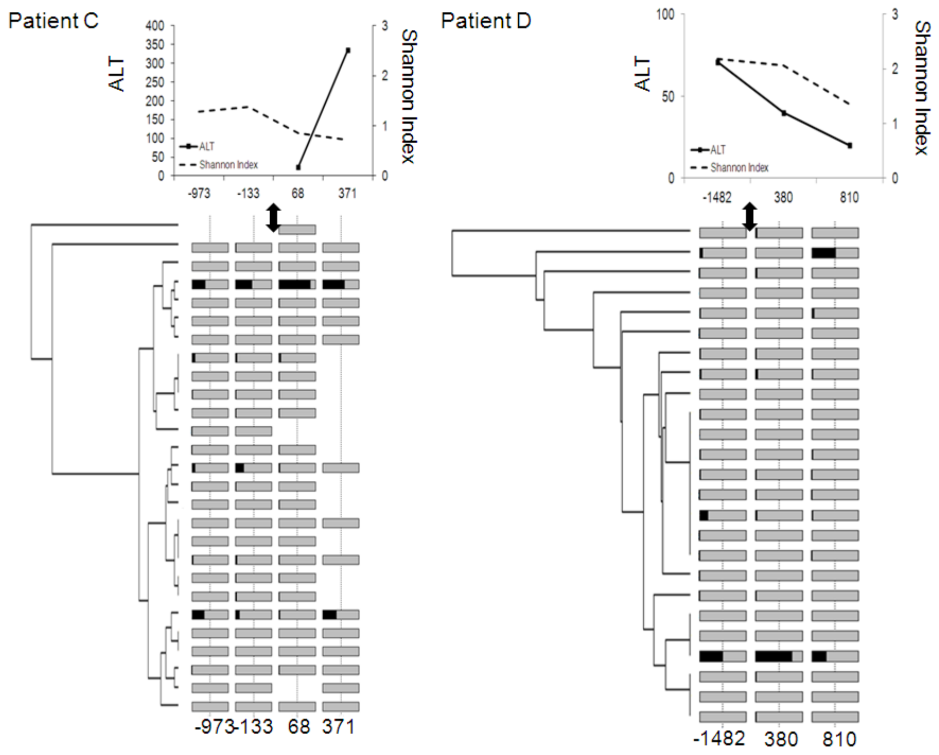

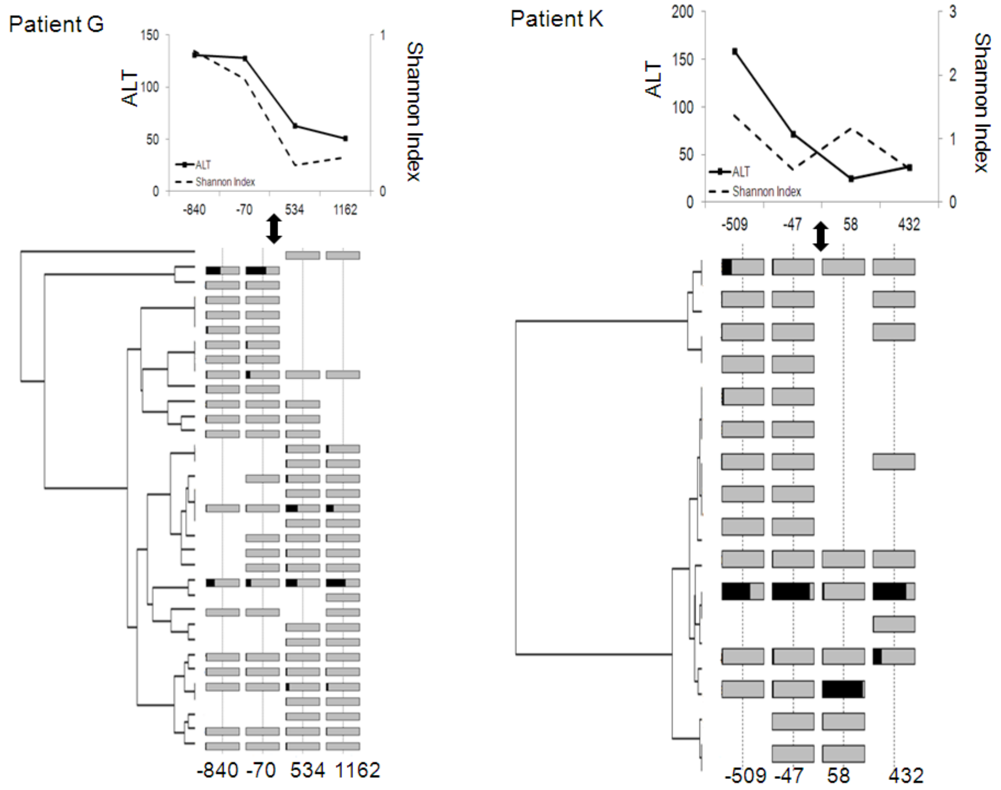
Patient G
ALT
Shannon Index
150
100
50
0
1
0
ALT
Shannon Index
-840
-70
534
1162
-840 -70 534 1162
Patient K
ALT
Shannon Index
200
150
100
50
0
3
2
1
0
ALT
Shannon Index
-509
-47
58
432
-509 -47 58 432

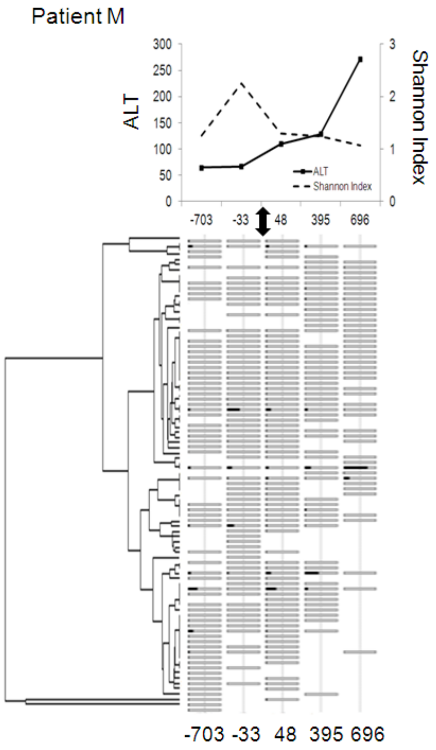
Patient M
ALT
Shannon Index
ALT
Shannon Index
-703
-33
48
395
696
-703 -33 48 395 696

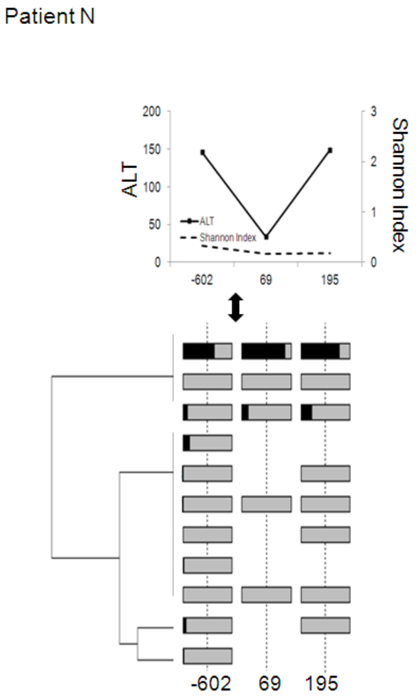
Patient N
ALT
Shannon Index
ALT
Shannon Index
-602
69
195
-602 69 195

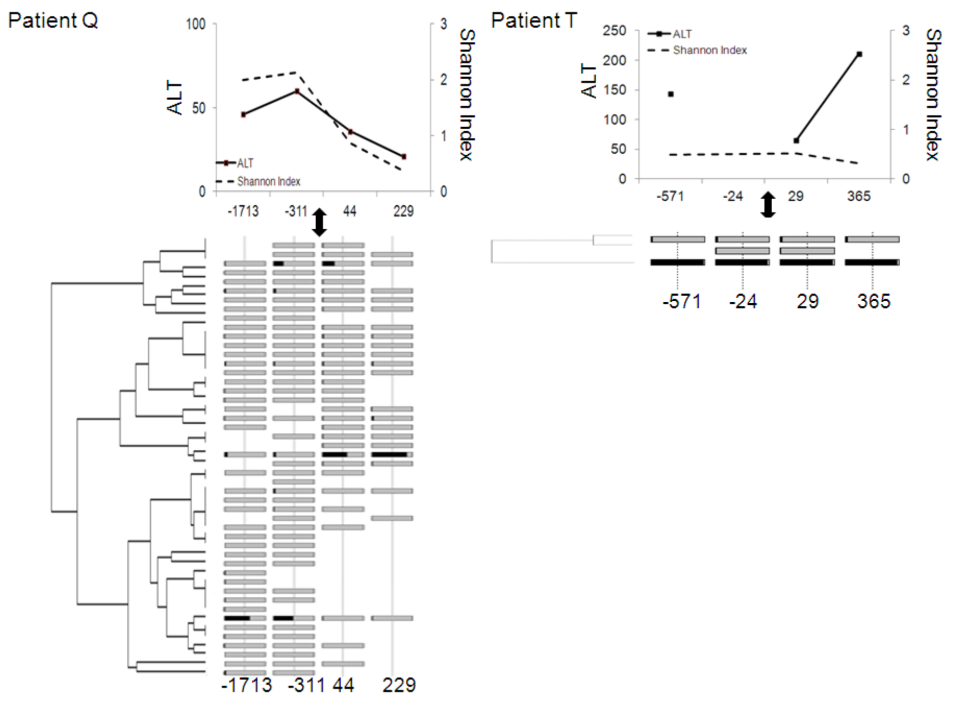

Supplementary Figure S1: NS3 quasispecies evolution and dynamics in liver transplant recipients.
